# A quality-improvement approach to urgent-care antibiotic stewardship for respiratory tract infections during the COVID-19 pandemic: Lessons learned

**DOI:** 10.1017/ice.2023.8

**Published:** 2023-12

**Authors:** Sharon K. Ong’uti, Maja Artandi, Brooke Betts, Yingjie Weng, Manisha Desai, Christopher Lentz, Ian Nelligan, David R. Ha, Marisa K. Holubar

**Affiliations:** 1 Vanderbilt University Medical Center, Nashville, Tennessee; 2 Express Care, Stanford Health Care, Stanford, California; 3 Department of Pharmacy, Stanford Health Care, Stanford, California; 4 Quantitative Sciences Unit, Stanford University School of Medicine; 5 Division of Pediatric Infectious Diseases, Stanford University School of Medicine, Stanford, California; 6 Primary Care and Population Health, Stanford University School of Medicine, Stanford, California; 7 Department of Quality, Patient Safety and Effectiveness, Stanford Health Care, Stanford, California; 8 Division of Infectious Diseases and Geographic Medicine, Stanford University School of Medicine, Stanford, California

## Abstract

**Objective:**

We investigated a decrease in antibiotic prescribing for respiratory illnesses in 2 academic urgent-care clinics during the coronavirus disease 2019 (COVID-19) pandemic using semistructured clinician interviews.

**Methods:**

We conducted a quality-improvement project from November 2020 to May 2021. We investigated provider antibiotic decision making using a mixed-methods explanatory design including interviews. We analyzed transcripts using a thematic framework approach to identify emergent themes. Our performance measure was antibiotic prescribing rate (APR) for encounters with respiratory diagnosis billing codes. We extracted billing and prescribing data from the electronic medical record and assessed differences using run charts, *p* charts and generalized linear regression.

**Results:**

We observed significant reductions in the APR early during the COVID-19 pandemic (relative risk [RR], 0.20; 95% confidence interval [CI], 0.17–0.25), which was maintained over the study period (*P* < .001). The average APRs were 14% before the COVID-19 pandemic, 4% during the QI project, and 7% after the project. All providers prescribed less antibiotics for respiratory encounters during COVID-19, but only 25% felt their practice had changed. Themes from provider interviews included changing patient expectations and provider approach to respiratory encounters during COVID-19, the impact of increased telemedicine encounters, and the changing epidemiology of non–COVID-19 respiratory infections.

**Conclusions:**

Our findings suggest that the decrease in APR was likely multifactorial. The average APR decreased significantly during the pandemic. Although the APR was slightly higher after the QI project, it did not reach prepandemic levels. Future studies should explore how these factors, including changing patient expectations, can be leveraged to improve urgent-care antibiotic stewardship.

Globally, more than two–thirds of antibiotics are prescribed in the outpatient setting.^1^ The CDC estimates that at least 30% of outpatient antibiotic prescriptions in the United States are unnecessary.^2^ Prior to the coronavirus disease 2019 (COVID-19) pandemic, urgent care centers (UCCs) had both the highest percentages of visits resulting in antibiotic prescriptions and the highest rates of inappropriate prescribing for respiratory tract infections across all healthcare settings,^3^ making them a priority target for stewardship interventions.^4^

Factors that influence antibiotic prescribing in the outpatient setting have been well described, including patient demand and variable clinician prescribing practices.^5^ It has been challenging to address many of these factors in UCCs^6,7^ due in part to the lack of a longitudinal provider–patient relationship.^8^ Like others, we observed a decrease in antibiotic prescribing in our UCCs during the COVID-19 pandemic without a direct stewardship intervention.^9–11^ We hypothesized that this decline may offer insights into how to better optimize antibiotic prescribing practices in UCCs.

We initiated a quality-improvement (QI) project aimed toward maintaining lower antibiotic prescribing rates (APRs) for encounters for respiratory complaints. As part of this project, we sought to describe the primary drivers of clinician’s antibiotic prescribing during the COVID-19 pandemic in 2 academic UCCs using semistructured clinician interviews.

## Methods

This project was conducted at 2 academic UCCs with 22 regular providers (13 physicians, 9 advance practice providers or APPs) and 23 staff who conduct >32,000 patient encounters per year. The clinics are same-day–access UCCs that primarily see patients for acute-care concerns in an urban setting in Santa Clara County, California. The project was part of a structured QI program^12,13^ and detailed project information is presented in the Supplementary Material using the Standards for Quality Improvement Reporting Excellence (SQUIRE) 2.0 guidelines^14^ (Supplementary Figures S1–S4 and Supplementary Surveys S1 and S2 online). We selected a mixed-methods sequential explanatory design to integrate a quantitative evaluation (see Supplementary Material online) followed by a qualitative assessment to gain insight into clinical practice.^15^

### Qualitative interviews

To understand clinician’s medical decision making regarding antibiotic prescribing during COVID-19, 1 team member (B.B.) who is a trained interviewer conducted semistructured interviews in March 2021. This team member was not known to the providers and was not part of the UCC or antimicrobial stewardship team. A standardized interview guide was developed using a consensus approach. We recruited UCC clinicians by email; participation was voluntary, and no compensation was provided. To protect clinicians’ privacy, no demographic data were collected. All interviews were 60 minutes long and were conducted virtually. Participants gave verbal consent prior to the interviews.

The interviewer used an appreciative inquiry approach,^16^ asking participants open-ended questions and exploring new ideas that emerged during the interview regarding changes in antibiotic prescribing practices during the pandemic (Supplementary Survey S2 online). We recorded and transcribed the interviews verbatim and analyzed them using a thematic framework approach designed to identify emergent standardized themes. Each transcription was independently reviewed and coded into key themes by 2 blinded investigators and adjudicated by a third investigator for stability, robustness, and interrater reliability. These themes were discussed as a group, and discrepancies were addressed resulting in the development of a combined revised thematic framework that captured the shared understanding.

### Performance measures

We calculated the antibiotic prescribing rate (APR) as the proportion of encounters in which an antibacterial drug (β-lactams, macrolides, lincosamides, sulfonamides, nitrofurans, nitroimidazoles, oxazolidinones, quinolones, tetracyclines, and fosfomycin) was prescribed.^11^ We extracted *International Classification of Disease, Tenth Revision* (ICD-10) codes and antibiotic data for all UCC encounters from the electronic medical record from January 2019 to December 2021. We used methodology from the *International Classification of Disease, Tenth Revision* (ICD-10) validated in UCCs^4^ that we modified to include COVID-19 ICD-10 codes^17^ to assign each encounter a disease category (gastrointestinal, genitourinary, skin, respiratory, and other) and a prescribing tier based on whether antibiotics are almost always (tier 1), sometimes (tier 2), or almost never (tier 3) indicated. For encounters with ICD-10 codes in multiple tiers, we assigned the lowest tier. For multiple ICD-10 codes within the same tier, we chose the first extracted ICD-10 code. We targeted the APR for respiratory tier 3 encounters because it represented encounters for which antibiotics were not indicated.

### Statistical analysis

We developed a run chart as well as a statistical process control chart (*p* chart) to monitor the respiratory tier 3 APR trend over time. The *p* chart was selected because it is used for binary data to track the proportion with an event for consecutive periods of time.^17^ This approach allowed the comparison of the periods before and after the change that were well defined and were specified prior to analyses.

A priori, we defined January–December 2019 as the “pre–COVID-19 pre-QI” period. We defined January 2020–March 2020 as the “peri–COVID-19 pre-QI” period due to the potential for unrecognized circulation of severe acute respiratory syndrome coronavirus 2 (SARS-CoV-2) during this time. We defined April 2020–December 2020 as the “COVID-19 pre-QI” period. Finally, we defined January 2021–May 2021 as the “COVID-19 QI” period and June 2021–December 2021 as the “COVID-19 post-QI” period.

A generalized linear regression model with log link was applied to assess the APR differences for these 4 periods compared to a baseline period that occurred before COVID-19 and before the QI project (January–December 2019), while adjusting for seasonality. More specifically, we applied a Poisson regression model by regressing the outcome (ie, the number of encounters with antibiotic prescriptions in given month) on the 5-level categorical variable representing the periods and a 4-level categorial variable representing seasons. The number of total encounters in each month was incorporated as an offset term. Risk ratios corresponding 95% confidence intervals and an overall *P* value for the parameter of interest over the studied periods were reported.

### Ethical considerations

This quality improvement project was deemed non–human-subjects research by the Stanford University School of Medicine Panel on Human Subjects in Medical Research.

## Results

### Qualitative interviews

**Fig. 1.**
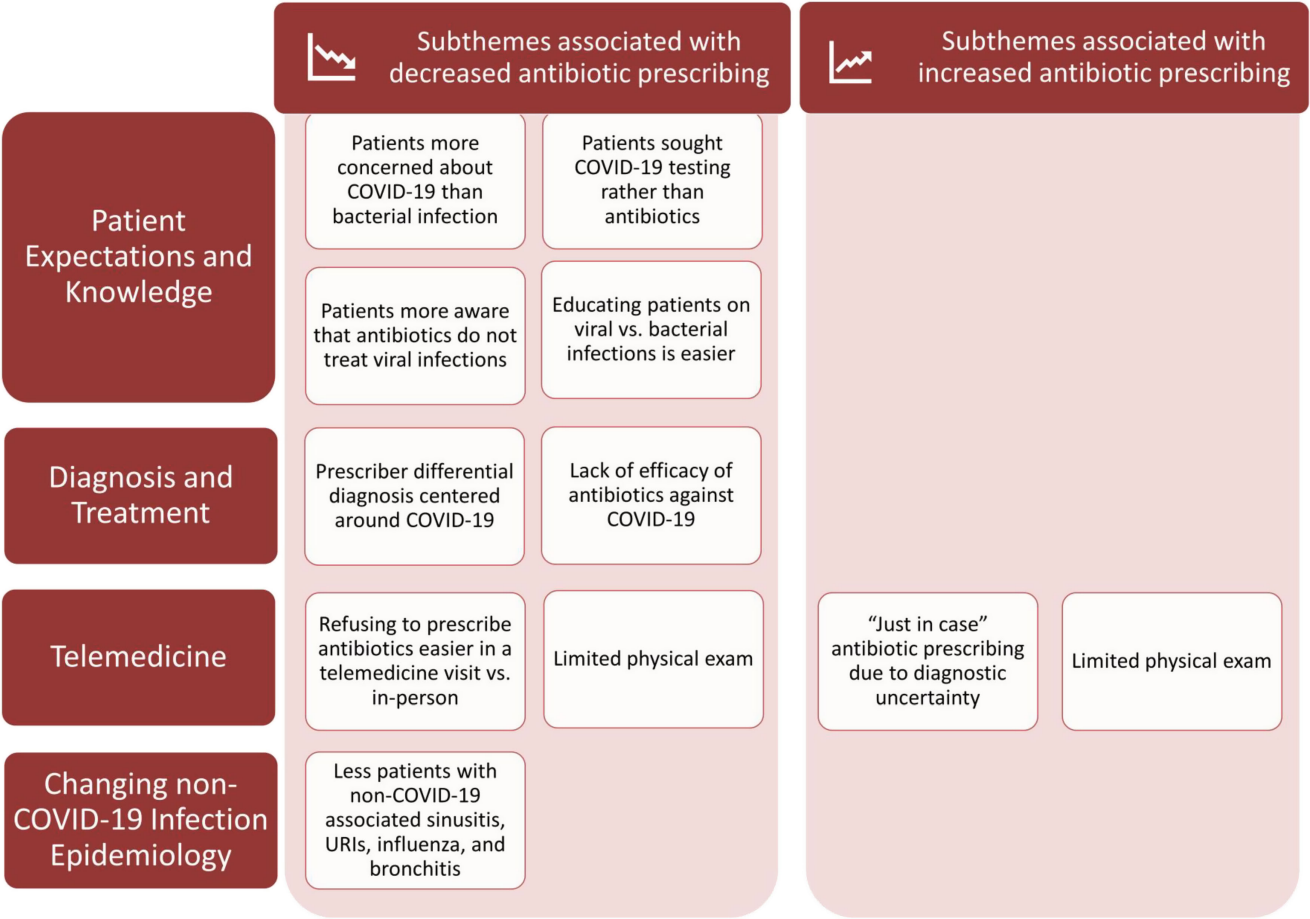
Summary of qualitative themes and subthemes the emerged from clinician interviews investigating antibiotic prescribing at academic urgent-care clinics during COVID-19.^23^

We interviewed all 12 clinicians who volunteered and categorized their responses into 4 major themes. Figure 1 and Table 1 demonstrate the COVID-19–related subthemes.

**Table 1.** Qualitative Themes and Illustrative Quotes From Semistructured Clinician Interviews

Theme Extracted from Survey Responses	Clinician Perspective
Patients more concerned about COVID-19 than bacterial infection	*There’s more focus on COVID and viral illnesses than on bacterial illnesses. So in a regular year that same viral illness can be misinterpreted as a bacterial infection, but this year I think the focus was on COVID so most patients were fine with having a negative COVID study and taking care of the symptoms on their own versus going for an antibiotic for their sinus symptoms.*
Patients sought COVID-19 testing rather than antibiotics	*The concerns that are on patient’s minds are not a bacterial infection, the concern is a COVID infection, so they’re not necessarily requesting antibiotics they’re requesting a COVID test. I think that has changed the demands on the provider and so they’re not pressured to prescribe antibiotics.*
Patients more aware that antibiotics do not treat viral infections	*I think in a way COVID-19 has helped us be able to educate patients about viral infections and help them understand by learning about viruses a little bit more, that an antibiotic is not going to treat it.*
Educating patients on viral vs bacterial infections is easier	*Because of COVID and the understanding now, patients are a little bit more receptive to the fact that they understand now what is viral, what is bacterial. Not everybody, but I think that with some patients, I think it’s easier to explain to them than before. It’s easier to have that conversation I feel now, than before because people are a little bit more aware about upper respiratory infections.*
**Diagnosis and treatment**	
Prescriber differential diagnosis centered around COVID-19	*It’s also possible that all of us as providers kind of got a little binary. It’s either COVID or it’s not COVID, it’s COVID or it’s not. I caught myself, my differentials were starting to narrow.*
Lack of efficacy of antibiotics against COVID-19	*Typically, if it wasn’t COVID if they came with those same symptoms they would probably be asking about antibiotics, but because with COVID I think they’re more concerned about the viral pathogen, and so they understand that antibiotics aren’t effective.*
**Telemedicine**	
Limited physical exam	*I would not prescribe an antibiotic I think via telemedicine without examining the patient. So yeah 100%, I think getting an exam, listening to the lungs, looking at the throat, and all of that would definitely be necessary for me to even consider prescribing an antibiotic.*
Refusing to prescribe antibiotics easier in a telemedicine visit vs in person	*If it’s a telemedicine visit it’s a little bit easier to say no to somebody who isn’t sitting right next to you.*
“Just in case” antibiotic prescribing due to diagnostic uncertainty	*Telehealth has created a whole new sort of opportunity for prescribing, especially “just in case medicines.” I don’t think this is what it is, but I think the consequences of me missing this diagnosis because I can’t actually see you or feel you or hear you is worse than me just giving you the antibiotics.*
**Changing non–COVID-19 infection epidemiology**	
Fewer patients with sinusitis, upper respiratory infections, influenza, bronchitis	*I feel like we’re seeing a lot less upper respiratory infections in general, far fewer cold and flu visits than we normally would. And it’s all the education available this year, I think that’s playing a big role in this. It could also be that people are not getting as sick this year. Staying inside, people are wearing masks, so there’s less people with upper respiratory infections this year.*

### Theme 1: Patient expectations and knowledge

Providers felt that during the COVID-19 pandemic, it was easier to educate patients about viral versus bacterial infections due to robust public health messaging. Providers expressed that their patients had a better understanding that COVID-19 is a viral infection that does not respond to antibiotics, leading to fewer antibiotic requests and making it easier to convince patients that antibiotics were not indicated when they were requested.

Providers also reported that the focus of clinic visits from both the provider and patient perspective shifted toward ruling out COVID-19, and away from a focus on common or seasonal respiratory concerns. Consequently, if the patient had a negative severe acute respiratory coronavirus virus 2 (SARS-CoV-2) test result, they were more receptive to symptomatic treatment and did not ask for antibiotics as they would have in the past.

Providers felt that when patients have historically received antibiotics for a ‘similar presentation,’ their expectation is that they will receive antibiotics again, and it may be challenging to address the patient’s demand. Providers felt that patient motivation for this included fear of complications if they do not receive antibiotics or an expectation that since they “paid” for the visit, they should receive an antibiotic.

Providers universally felt that patients are more likely to report that they are satisfied with their provider if they prescribed antibiotics. Providers worried that when supportive care alone is offered, patients may feel disappointed. Providers reported institutional pressure to have higher patient satisfaction scores, so they focus on ensuring that the patient is pleased with the outcome of the visit.

### Theme 2: Diagnosis and treatment

During COVID-19, providers reported that the differential diagnoses often centered around COVID-19 and may have been a factor in decreasing antibiotic utilization.

Providers reported that diagnostic uncertainty coupled with concern for a missed infection, (eg, a patient with a cough who appears clinically ill but has a chest radiograph not consistent with bacterial pneumonia) might contribute to suboptimal antibiotic prescribing. Several providers felt that they may also prescribe antibiotics as ‘a last resort’ to patients with persistent symptoms despite having tried supportive treatments (eg, for sinusitis). Providers also indicated that when they were under time constraints, they prescribed antibiotics more frequently instead of spending time counseling a patient regarding why antibiotics were not indicated.

Providers felt that the lack of evidence-based guidelines for certain diagnoses as well as incomplete adherence to guidelines, even when they are available, contribute to antibiotic overuse. Most agreed that having a consensus on protocols would be helpful in unifying practice and consequently reducing overprescribing. Providers reported that individual training and past experience can either lead to an increase or decrease in antibiotic prescribing.

### Theme 3: Telemedicine

Clinicians’ opinions regarding the impact of telemedicine on antibiotic prescribing were mixed. Some providers reported that telemedicine made it challenging to thoroughly evaluate patients, which could lead to both antibiotic over- and underprescribing. For example, a patient being evaluated for cough coupled with a limited physical exam may be more likely to be prescribed antibiotics to avoid a poor outcome just in case an actual pneumonia is missed. Conversely, some providers felt that they would not prescribe an antibiotic via telemedicine, suggesting that the physical exam influenced their antibiotic prescribing decision making. Some providers also felt that it was easier to refuse antibiotics in a telemedicine compared to an office visit.

### Theme 4: Changing non–COVID-19 epidemiology of infections

Providers felt that they were seeing fewer patients with upper respiratory tract infections, sinusitis, and influenza during the COVID-19 pandemic.

### Performance measures

**Fig. 2.**
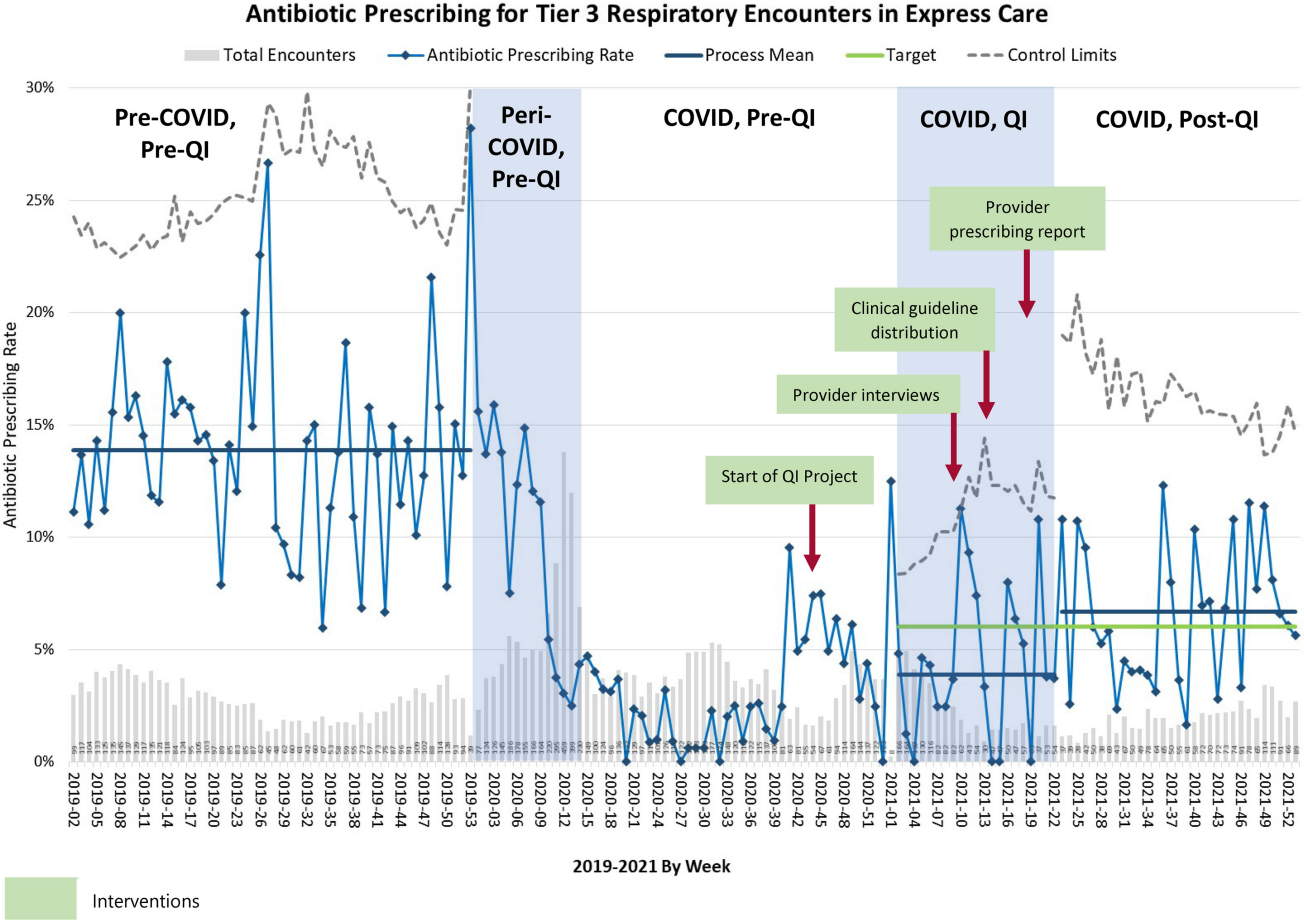
P chart of antibiotic prescribing for respiratory tier 3 encounters and summary of project phases.

The provider-specific antibiotic prescribing data revealed that all clinicians prescribed less during COVID-19 compared to the pre–COVID-19 period (Supplementary Fig. S4 online). Our project annotated control chart is shown in Figure 2. The average tier 3 respiratory APR was 14% before the COVID-19 pandemic, 3% during the COVID-19 pandemic but before the QI project, 4% during the QI project, and 7% after the QI project.

After adjusting for seasonality during the period of 2019 to 2021, we observed that significant reductions in APR occurred early during the COVID-19 pandemic (relative risk [RR], 0.20; 95% confidence interval [CI], 0.17–0.25), and this reduction was maintained over the study period (RR during the project, 0.26; 95% CI, 0.20–0.34; RR after the project, 0.51; 95% CI, 0.41–0.61; *P* < .001) (Table 2). The APR in the post-QI phase was still 49% lower relative to the pre-COVID-19 period (RR, 0.51; 95% CI, 0.41–0.62).

**Table 2.** Differences in Antibiotic Prescribing Rates by Period

Date	Period	Risk Ratio (95% CI)	*P* Value
			<.001
January–December 2019	Pre–COVID-19, Pre-QI	Ref	
January–March 2020	Peri–COVID-19, Pre-QI	0.51 (0.43–0.60)	
April–December 2020	COVID-19, Pre-QI	0.20 (0.17–0.25)	
January–May 2021	COVID-19, QI	0.26 (0.20–0.34)	
June–December 2021	COVID-19, Post-QI	0.51 (0.41–0.61)	

Note. CI, confidence interval; QI, quality improvement project; peri–COVID-19, possible unrecognized circulation of SARS-CoV-2.

## Discussion

Several factors likely contributed to a sustained reduction in tier 3 respiratory APR during the COVID-19 pandemic. These factors included perceived changes in patient knowledge and expectations about the management of respiratory viral illnesses, the dominance of COVID-19 on the differential diagnosis for patients with respiratory symptoms, a switch to telemedicine-based encounters, and changing communicable disease epidemiology.

The collective dominance of COVID-19 on the patient and clinician’s minds appeared to shift the focus of encounters for respiratory symptoms to a specific diagnosis. Before the pandemic, many patients with respiratory complaints were given a nonspecific diagnosis (eg, “likely viral respiratory illness”), which may have been unsatisfying to some patients. In contrast, during the pandemic, the conversation shifted to making or “ruling out” a laboratory-based COVID-19 diagnosis. If COVID-19 testing was positive, the conversation centered around test-result interpretation, anticipatory guidance for when to seek emergency medical care, and how to mitigate the risk of transmitting the virus to others, not that antibiotics were not indicated.

Clinicians also felt that it was easier to counsel patients regarding COVID-19 management because their patients’ understanding of this infection was more nuanced than for other respiratory viral infections, which may have been due in part to robust public health messaging. Even early in the pandemic, these public health campaigns emphasized the viral etiology of COVID-19, how to manage symptoms at home, and when to seek medical care. Notably, the messaging did not include antibiotics. Public health campaigns may have been more impactful during COVID-19 because of their scope and the public’s hunger for any information, especially early in the pandemic.^18^ Ultimately, this improved understanding of COVID-19 may have averted patient’s requests or truncated conversations about antibiotics.

Clinicians expressed opposing opinions regarding the impact of telemedicine on antibiotic prescribing. Many felt that the lack of a physical exam increased diagnostic uncertainty, but they differed on how this would impact antibiotic prescribing. Many felt that denying a patient’s request for antibiotics would be easier in telemedicine than in person, alluding to tense in-person conversations.^8^ The UCC clinicians’ lack of experience with telemedicine early in the pandemic may have contributed to these mixed opinions. Nevertheless, we previously reported a similar decrease in APR for UCC telemedicine and clinic encounters for respiratory conditions during COVID-19.^11^ Understanding the impact of telemedicine on the clinician–patient relationship and tracking APR in telemedicine and clinic encounters separately will continue to be important for ongoing outpatient stewardship efforts.

Our UCC clinicians cited many of the same conventional factors known to influence antibiotic-prescribing for respiratory illnesses before the COVID-19 pandemic including diagnostic uncertainty, patient satisfaction, and time constraints.^19–21^ As the pandemic evolves and other respiratory viruses circulate with SARS-CoV-2, these persistent factors may lead to a resurgence of inappropriate antibiotic prescribing for other viral respiratory illnesses and/or COVID-19. In fact, although the overall low respiratory APR was generally maintained over the study period, antibiotic prescribing increased after our QI project ended ∼1.5 years into the pandemic despite the implementation of sustaining measures. The reasons for this increase are unclear; possibilities include the Hawthorne effect,^22^ increased variation in clinical presentations seen as the local respiratory virus epidemiology changed, and a change in real or perceived patient pressures as attention to “ruling out” or managing COVID-19 lessened.

Our project had several limitations. First, this was a single-center project at an academic health system, and our results may not be comprehensive or generalizable to alternate settings. Second, we did not collect demographic data for the clinicians we interviewed to maintain their privacy. Third, we used encounter-level billing data to identify targeted tier 3 respiratory encounters for our process metrics, which may not have accurately reflected everything addressed during the clinic visit or the provider’s rationale if antibiotics were prescribed. However, most encounters (∼88%) had 1–2 associated ICD-10 (data not shown), and we focused on rates over time. Fourth, our project did not overtly include patient perspectives.

In conclusion, we observed a sustained reductions in the tier 3 respiratory APRs at 2 UCCs during the COVID-19 pandemic that were likely driven by multiple factors, including an increased public understanding of the symptomatic management of COVID-19 as well as the impact of a specific diagnosis for patients presenting with respiratory complaints. All UCC providers prescribed fewer antibiotics for respiratory encounters during the COVID-19 pandemic, even though most providers surveyed reported that their antibiotic prescribing behaviors had not changed during the pandemic. The impact of COVID-19 on antibiotic prescribing was pervasive, and the clinicians’ behavior change was unintentional, which suggests that different and more creative interventions designed to maintain this change may be needed. Rapid diagnostics for other respiratory viruses and extending public health and healthcare system messaging to reinforce the lack of efficacy of antibiotics against all respiratory viral pathogens, including COVID-19, could affect outpatient stewardship efforts and build on gains made in reducing suboptimal antibiotic prescribing during the pandemic.

## Supplementary material

For supplementary material accompanying this paper visit https://doi.org/10.1017/ice.2023.48. click here to view supplementary material

## References

[1] Hersh AL , King LM , Shapiro DJ , Hicks LA , Fleming-Dutra KE. Unnecessary antibiotic prescribing in US ambulatory care settings, 2010–2015. Clin Infect Dis 2021;72:133–137. 32484505 10.1093/cid/ciaa667 PMC9377284

[2] Sanchez GV , Fleming-Dutra KE , Roberts RM , Hicks LA. Core elements of outpatient antibiotic stewardship. Morb Mortal Wkly Rep Recomm Rep 2016;65:1–12. 10.15585/mmwr.rr6506a1 27832047

[3] Palms DL , Hicks LA , Bartoces M , et al. Comparison of antibiotic prescribing in retail clinics, urgent care centers, emergency departments, and traditional ambulatory care settings in the United States. JAMA Intern Med 2018;178:1267–1269. 30014128 10.1001/jamainternmed.2018.1632 PMC6142958

[4] Stenehjem E , Wallin A , Fleming-Dutra KE , et al. Antibiotic prescribing variability in a large urgent care network: a new target for outpatient stewardship. Clin Infect Dis 2020;70:1781–1787. 31641768 10.1093/cid/ciz910 PMC7768670

[5] Zetts RM , Stoesz A , Garcia AM , et al. Primary care physicians’ attitudes and perceptions towards antibiotic resistance and outpatient antibiotic stewardship in the USA: a qualitative study. BMJ Open 2020;10:e034983. 10.1136/bmjopen-2019-034983 PMC7365421 32665343

[6] Durkin MJ , Jafarzadeh SR , Hsueh K , et al. Outpatient antibiotic prescription trends in the United States: a national cohort study. Infect Control Hosp Epidemiol 2018;39:584–589. 29485018 10.1017/ice.2018.26 PMC7967296

[7] Buehrle DJ , Wagener MM , Nguyen MH , Clancy CJ. Trends in outpatient antibiotic prescriptions in the United States during the COVID-19 pandemic in 2020. JAMA Network Open 2021;4:e2126114. 34550387 10.1001/jamanetworkopen.2021.26114 PMC8459187

[8] Kohut MR , Keller SC , Linder JA , et al. The inconvincible patient: how clinicians perceive demand for antibiotics in the outpatient setting. Fam Pract 2020;37:276–282. 31690948 10.1093/fampra/cmz066

[9] Niemenoja O , Taalas A , Taimela S , Bono P , Huovinen P , Riihijärvi S. Time series analysis of the incidence of acute upper respiratory tract infections, COVID-19 and the use of antibiotics in Finland during the COVID-19 epidemic: a cohort study of 833,444 patients. BMJ Open 2022;12:e046490. 10.1136/bmjopen-2020-046490 PMC8804308 35105608

[10] King LM , Lovegrove MC , Shehab N , et al. Trends in US outpatient antibiotic prescriptions during the coronavirus disease 2019 pandemic. Clin Infect Dis 2021;73:e652–e660. 33373435 10.1093/cid/ciaa1896 PMC7799289

[11] Ha D , Ong’uti S , Chang A , et al. Sustained reduction in urgent care antibiotic prescribing during the coronavirus disease 2019 pandemic: an academic medical center’s experience. Open Forum Infect Dis 2022;9:ofab662. 35111874 10.1093/ofid/ofab662 PMC8802794

[12] Realizing Improvement through Team Empowerment (RITE). Standford Medicine website. https://med.stanford.edu/rsc/education.html. Accessed January 17, 2022.

[13] Larson DB , Mickelsen LJ , Garcia K. Realizing Improvement through Team Empowerment (RITE): a team-based, project-based multidisciplinary improvement program. Radiographics 2016;36:2170–2183. 27831843 10.1148/rg.2016160136

[14] Ogrinc G , Davies L , Goodman D , Batalden P , Davidoff F , Stevens D. Standards for quality improvement reporting excellence 2.0: revised publication guidelines from a detailed consensus process. J Surg Res 2016;200:676–682. 26515734 10.1016/j.jss.2015.09.015

[15] Ivankova NV , Creswell JW , Stick SL. Using mixed-methods sequential explanatory design: from theory to practice. Field Methods 2006;18:3–20.

[16] Bushe GR. Advances in appreciative inquiry as an organization development intervention. Org Devel J 1995;13:14–14.

[17] Mohammed MA , Worthington P , Woodall WH. Plotting basic control charts: tutorial notes for healthcare practitioners. BMJ Qual Saf 2008;17:137–145. 10.1136/qshc.2004.012047 18385409

[18] Lang R , Benham JL , Atabati O , et al. Attitudes, behaviours and barriers to public health measures for COVID-19: a survey to inform public health messaging. BMC Public Health 2021;21:1–15. 33882896 10.1186/s12889-021-10790-0 PMC8058588

[19] Dempsey PP , Businger AC , Whaley LE , Gagne JJ , Linder JA. Primary care clinicians’ perceptions about antibiotic prescribing for acute bronchitis: a qualitative study. BMC Fam Pract 2014;15:1–10. 25495918 10.1186/s12875-014-0194-5 PMC4275949

[20] Linder JA , Doctor JN , Friedberg MW , et al. Time of day and the decision to prescribe antibiotics. JAMA Internal Med 2014;174:2029–2031. 25286067 10.1001/jamainternmed.2014.5225 PMC4648561

[21] Coenen S , Francis N , Kelly M , et al. Are patient views about antibiotics related to clinician perceptions, management and outcome? A multicountry study in outpatients with acute cough. PLoS One 2013;8:e76691. 24194845 10.1371/journal.pone.0076691 PMC3806785

[22] Linder JA , Meeker D , Fox, CR , et al. Effects of behavioral interventions on inappropriate antibiotic prescribing in primary care 12 months after stopping interventions. JAMA 2017;318:1391–1392. 29049577 10.1001/jama.2017.11152 PMC5818848

[23] Betts B , Holubar M , Ha D , et al. 160. Urgent-care prescriber perspectives on antibiotic prescribing during the COVID-19 pandemic. Open Forum Infect Dis 2021;8 suppl 1:S190–S191.

