# Supplementary material for: A quality-improvement approach to urgent-care antibiotic stewardship for respiratory tract infections during the COVID-19 pandemic: Lessons learned
Source: Infect Control Hosp Epidemiol. Author manuscript; Available in PMC 2024 Feb 16. (PMC10445104; doi:10.1017/ice.2023.8)
Supplement: Ong'uti et al. Supplementary Material [file NIHMS1908974-supplement-1.docx]

**SUPPLEMENTARY MATERIAL**

| **Table of Contents** | **Page number(s)** |
| --- | --- |
| Supplemental Methods and Results | 2 |
| Supplemental Figure S1. The Project Progress Scale | 4 |
| Supplemental Figure S2. Key Drivers associated with antibiotic prescribing | 5 |
| Supplemental Figure S3. Cause and effect diagram | 6 |
| Supplemental Figure S4. Clinician prescribing rates | 7 |
| Survey S1: Provider Quantitative Questionnaire | 8 |
| Survey S2: Provider Qualitative Survey | 9 |

**Supplemental Methods and Results**
The following quality improvement project information is presented using the Standards for Quality Improvement Reporting Excellence (SQUIRE) 2.0 guidelines.^1^

**Project team**

We assembled a multidisciplinary team of individuals who represented different components in the care process including the UCC medical director, an antimicrobial stewardship program (ASP) medical director, a family medicine attending, an infectious diseases pharmacist, an infectious diseases stewardship fellow, an administrative pharmacy resident and the UCC nurse manager.

Project advancement was monitored using a progress scale. A modest improvement corresponded to a score of at least 3.0 (Figure S1). The guided program enabled teams to set and achieve systematic goals throughout the process and receive regular feedback from the course directors.

**Observing the Initial State**

*Please see description of the clinical setting in the manuscript*

*Quantitative survey*

An initial quantitative survey was given to the 22 regular providers (13 physicians and 9 advance practice providers). Questions assessing clinical management of respiratory tract infections were adapted from published literature^2-4^ and we added questions to assess the influence of COVID-19 on antibiotic decision-making (Supplemental Table S1). A printed copy of the survey was disseminated to all full-time prescribers including physicians and APPs by the UCC Medical Director in January 2021. The findings of this survey helped develop a cause-and-effect and key driver diagram to identify factors that directly impact antibiotic prescribing (Figure S2 and Figure S3.)

*Results of quantitative survey*

Most of the providers completed the survey (91% response rate). The majority (95%) acknowledged that inappropriate antibiotic prescribing is a problem in the US, however only 15% saw it as a problem in their own practice. Only 25% of the providers thought that COVID-19 changed their antibiotic prescribing practices for patients with respiratory infections. Most providers reported that they were more likely to prescribe antibiotics for an upper respiratory infection if the patient had repeated visits for the same problem (85%) or if they seemed ‘sick’ (65%).

**Key Drivers**

Using the survey results, interviews, and our collective experience, we identified the following factors that influenced antibiotic prescribing: patient satisfaction, access to medical decision-making tools, standardization of treatment of upper respiratory infections, data transparency to compare prescribing patterns between providers and addressing time management to reduce the duration needed for patient counseling (Figure S2).

**Interventions**

*Engagement of providers*: We presented an overview and progress report of this QI project to the UCC providers during monthly provider meetings in February 2021 and March 2021.

*Syndrome specific guidelines*: We developed institution specific clinical guidelines for common symptoms of upper respiratory tract infections (sinus pressure, cough) and disseminated them in April 2021 during the meetings and by email. The guidelines were also posted in every room for quick reference.

*Provision of provider-specific antibiotic prescribing data*: All 22 regular UCC providers received confidential feedback on their individual Tier 3 respiratory APR compared to their peers through email in May 2021 (Figure S4).

Supplemental Figure S1. The Project Progress Scale

Supplement Figure S2. Key Drivers associated with antibiotic prescribing

Supplemental Figure S3. Cause and effect diagram

Supplemental Figure S4. Clinician prescribing rates

Survey S1: Provider Quantitative Questionnaire

|  | **Questions** | **Strongly agree** | **Agree** | **Neither agree nor disagree** | **Disagree** | **Strongly disagree** |
| --- | --- | --- | --- | --- | --- | --- |
| 1 | Inappropriate antibiotic prescribing is a problem in the United States |  |  |  |  |  |
| 2 | Inappropriate antibiotic prescribing is a problem for my colleagues in Express Care |  |  |  |  |  |
| 3 | Inappropriate antibiotic prescribing is a problem in my personal practice |  |  |  |  |  |
| 4 | I am aware of clinical tools that help my decision to prescribe antibiotic |  |  |  |  |  |
| 5 | Clinical tools for the prescribing of antibiotics are helpful |  |  |  |  |  |
| 6 | Easily accessible institution specific guidelines for antibiotic prescribing would be useful |  |  |  |  |  |
| 7 | Information for patients on antibiotics would be very useful |  |  |  |  |  |
| 8 | Developing standardized workflows for the treatment of upper respiratory tract infections would be very helpful |  |  |  |  |  |
| 9 | COVID-19 has changed my antibiotic prescribing practices for patients with respiratory infections |  |  |  |  |  |
| 10 | I am more likely to prescribe antibiotics for an Upper respiratory tract infection (Please select all that apply)   1. When I perceive that the patient wants antibiotics 2. When I am running late 3. When the patient had repeated visits for the same problem 4. When I am seeing the patient on a virtual visit 5. When I am not sure what is going on and am worried that the patient will get worse without a prescription for antibiotics. 6. When I am worried about patient satisfaction if I don’t prescribe antibiotics 7. Other | | | | | |

Survey S2: Provider Qualitative Survey

| 1. What are your thoughts on appropriateness of antibiotic prescribing for respiratory infections?  a. If stated as inappropriate: Is this a challenge in the United States? a challenge for my colleagues in Express Care? a challenge in my personal practice?  i. Follow up: What are your insights as to WHY antibiotics are inappropriately prescribed?  2. What clinical tools (like guidelines, Sanford, or other references) do you use to help you make antibiotic prescribing decisions for patients with respiratory infections? Do you use more than one?  a. Follow up: How/When do you use them?  b. What information they are looking up (Dosing, duration, etc.)?  c. Why do you use these resources?  d. How often do you use these resources? Every day? Every time?  3. What circumstances might lead one to prescribe an antibiotic for respiratory infections even when it may not be clinically indicated?  a. Follow up: Are there any resources or support that could be helpful to refrain from prescribing the antibiotic in such circumstances?  4. Has COVID-19 changed your antibiotic prescribing practices for patients with respiratory infections? If yes, how?  5. Do you feel that an interaction via telemedicine, versus in person, changes your antibiotic prescribing practices for patients with respiratory infections? If yes, how?  6. In your opinion, have COVID-19 and telemedicine affected patient expectations of antibiotics in the setting of a respiratory infection? If yes, how?  7. “We’ve noted a decline in antibiotic prescriptions for patients with respiratory illnesses evaluated by Express Care during the pandemic. Do you have any ideas re: why that might be?”  8. Can you think of ways that the clinic workflow could be changed to reduce inappropriate antibiotic prescribing for respiratory infections?  9. What clinical tools would be most helpful to you?  a. Follow up: Bring up Decision support tools (smart sets), Guidelines  b. Follow up: What clinical syndromes (e.g. UTI, CAP, etc.) are the most important  c. Follow up (Guidelines): What format is preferred (electronic, posted, built into decision support, etc.)  d. How important do you think embedding decision support tools in EPIC is in order to effect change?  10. Is there anything other suggestions you have that would improve antibiotic prescribing in express care |
| --- |

**References**

1. Ogrinc, G., et al., *Standards for QUality Improvement Reporting Excellence 2.0: revised publication guidelines from a detailed consensus process.* Journal of surgical research, 2016. **200**(2): p. 676-682.

2. Mustafa, M., et al., *Managing expectations of antibiotics for upper respiratory tract infections: a qualitative study.* The Annals of Family Medicine, 2014. **12**(1): p. 29-36.

3. Dempsey, P.P., et al., *Primary care clinicians’ perceptions about antibiotic prescribing for acute bronchitis: a qualitative study.* BMC family practice, 2014. **15**(1): p. 1-10.

4. Zetts, R.M., et al. *Primary care physicians’ attitudes and perceptions towards antibiotic resistance and antibiotic stewardship: a national survey*. in *Open forum infectious diseases*. 2020. Oxford University Press US.
