# Supplementary figures and images for: A quality-improvement approach to urgent-care antibiotic stewardship for respiratory tract infections during the COVID-19 pandemic: Lessons learned
Source: Infect Control Hosp Epidemiol. Author manuscript; Available in PMC 2024 Feb 16. (PMC10445104; doi:10.1017/ice.2023.8)

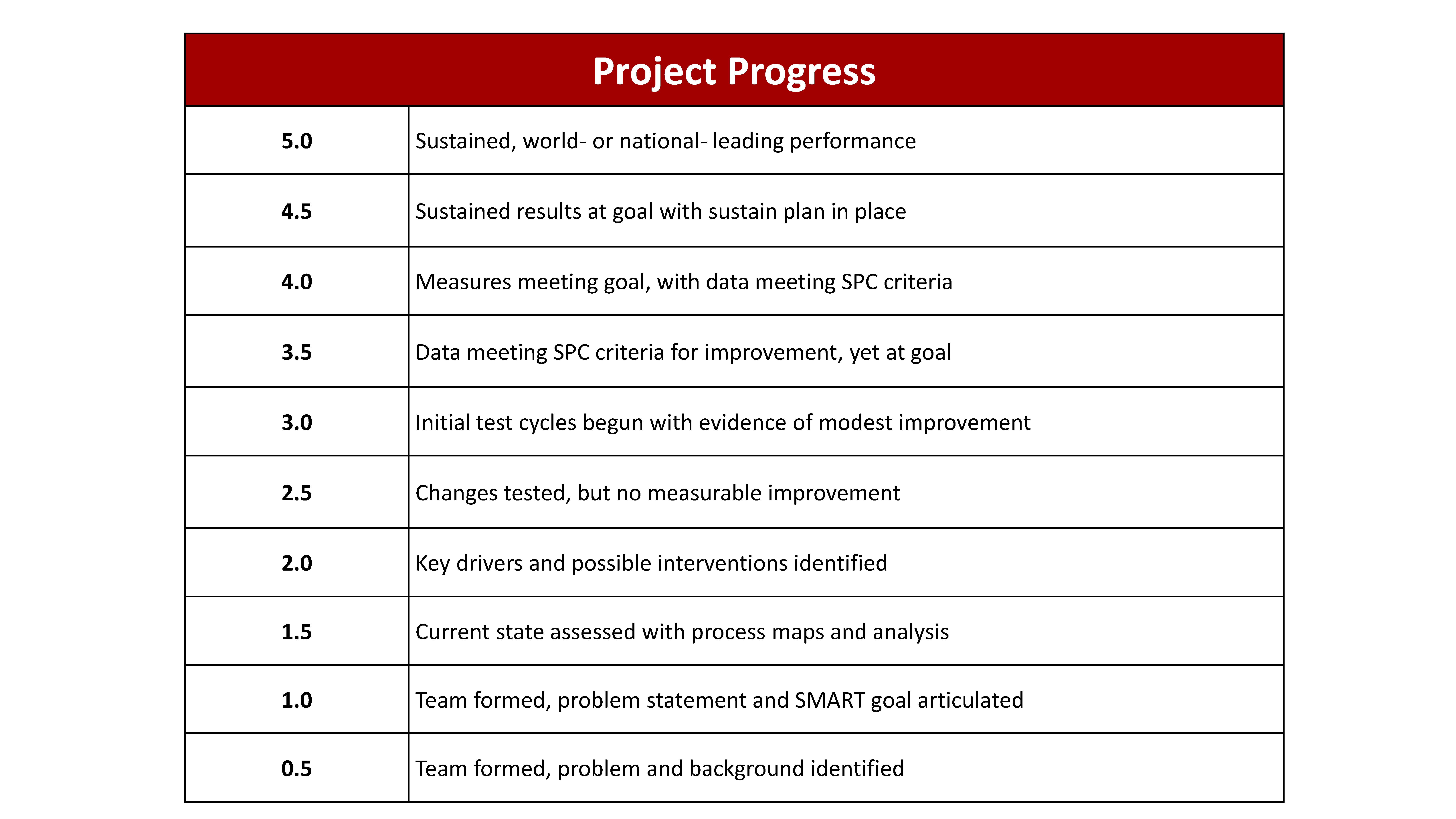

Supplement: Supplemental figure S1 [file NIHMS1908974-supplement-Supplemental_figure_S1.tif]

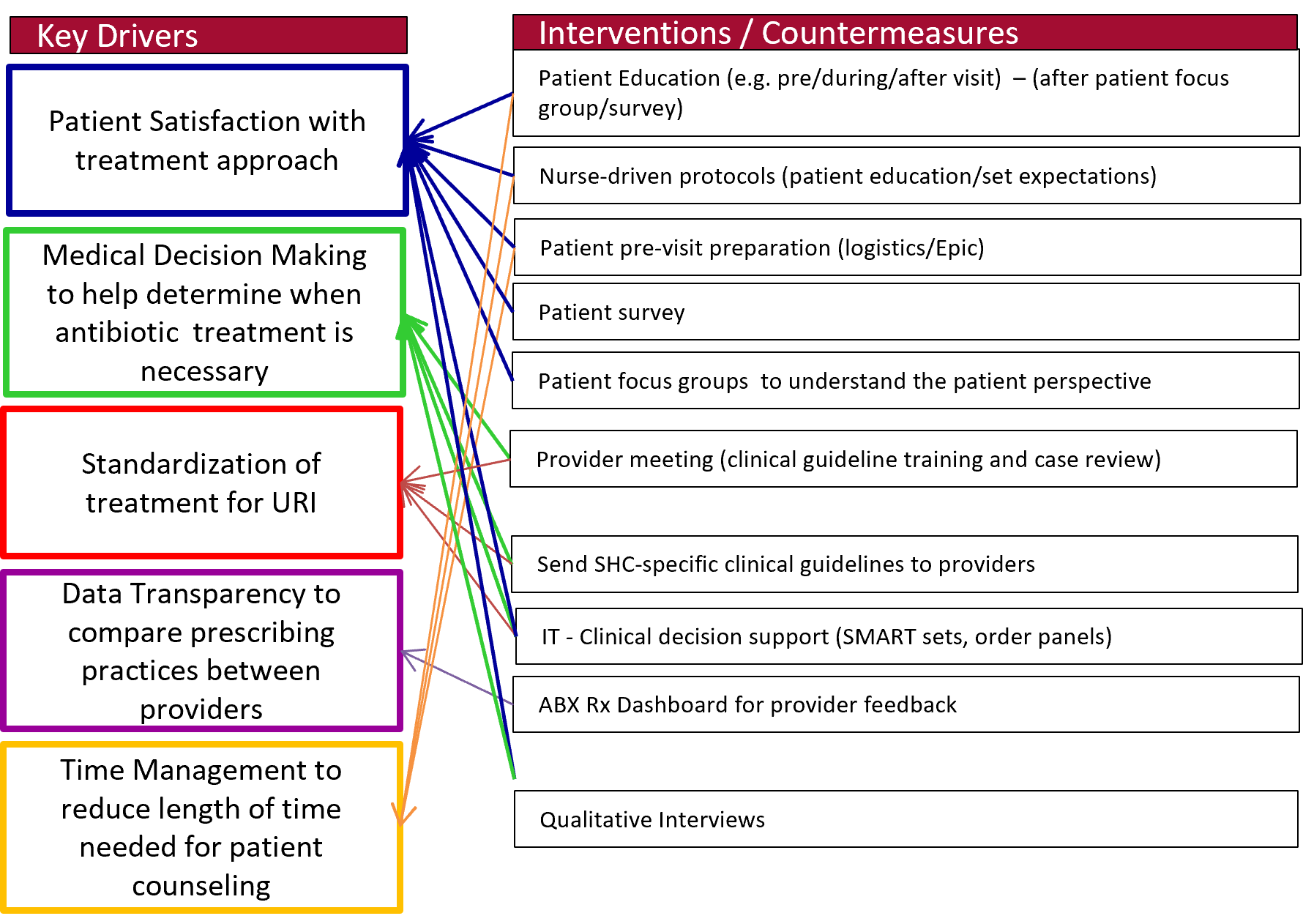

Supplement: Supplemental figure S2 [file NIHMS1908974-supplement-Supplemental_figure_S2.tif]

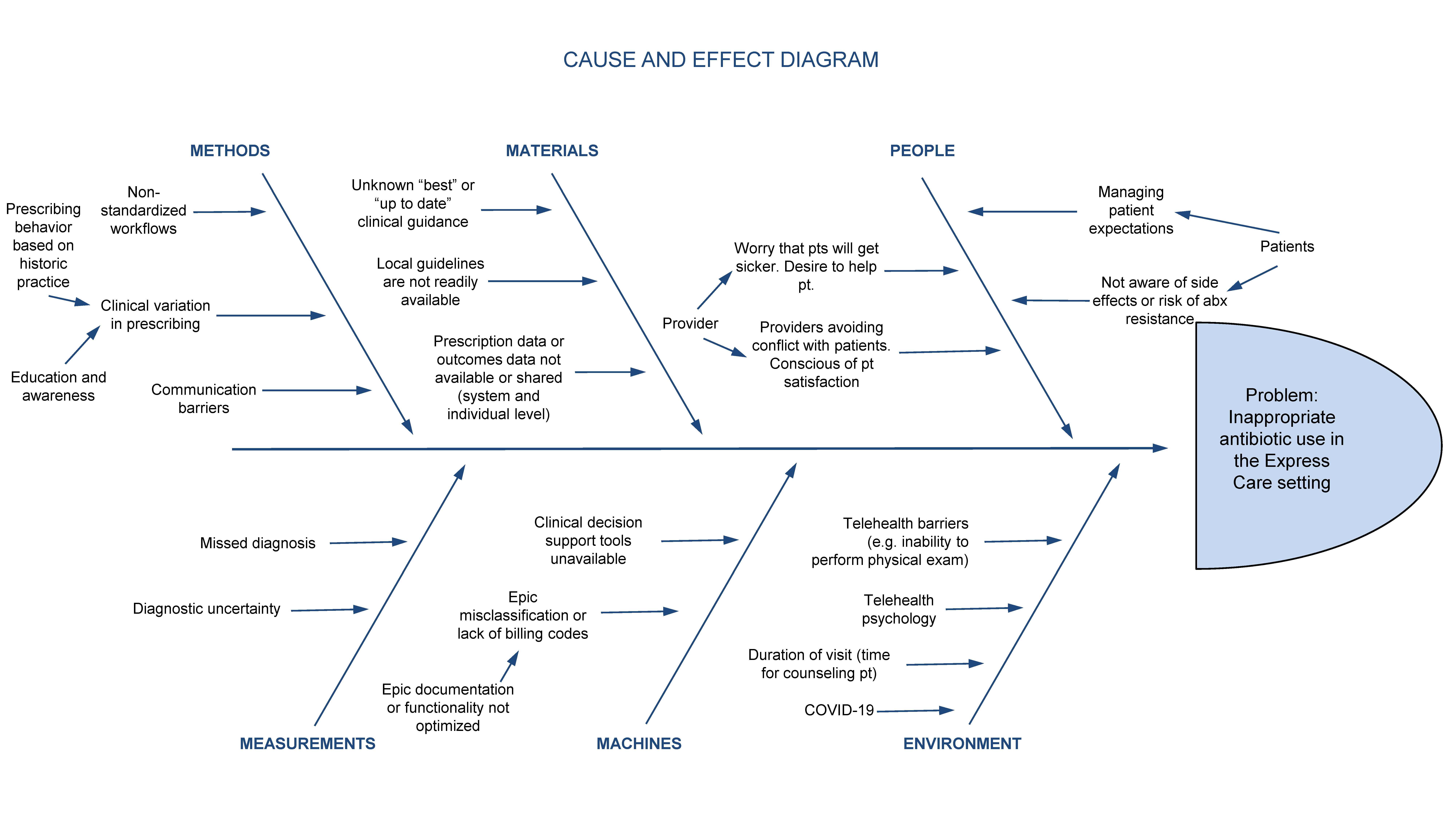

Supplement: Supplemental figure S3 [file NIHMS1908974-supplement-Supplemental_figure_S3.tiff]

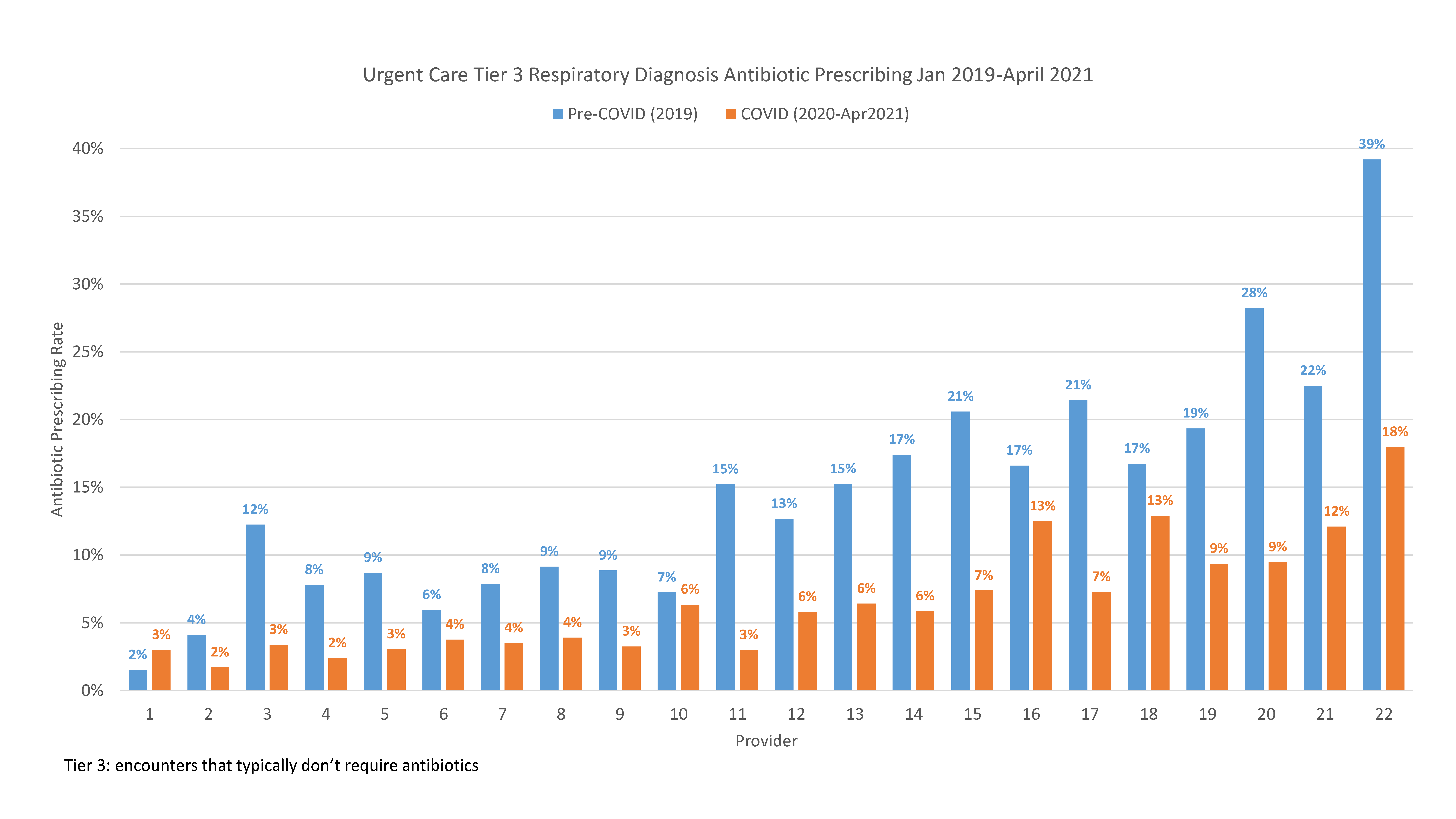

Supplement: Supplemental figure S4 [file NIHMS1908974-supplement-Supplemental_figure_S4.tif]
